# The effects of acupuncture on psychological symptoms in patients with insomnia: study protocol for a randomized controlled trial

**DOI:** 10.1186/s13063-022-06078-2

**Published:** 2022-02-15

**Authors:** Lifen Wang, Ruisen Wang, Yanling Yao, Xue Bai, Gang Sheng

**Affiliations:** 1 Department of Acupuncture and Moxibustion, Shaanxi Provincial Hospital of Chinese Medicine, Xi’an, 710003 Shaanxi China; 2 Shaanxi University of Traditional Chinese Medicine, Xianyang, 712046 Shaanxi China

**Keywords:** Acupuncture, Insomnia, Anxiety, Depression, Quality of life

## Abstract

**Background:**

Insomnia is a common sleep-related condition that includes dissatisfaction with sleep quality, difficulty in initiating or maintaining sleep, and early morning waking. Insomnia can affect daytime functioning by causing fatigue, depression, and anxiety. Medications are the most common method for the management of insomnia but can cause adverse effects, including psychological and physical dependence, residual daytime sedation, and cognitive impairment. Acupuncture is a common traditional Chinese therapy. It has been used in the treatment of insomnia, depression, and anxiety in China. However, there are no high-quality studies focusing on acupuncture for insomnia, especially for depression and anxiety due to insomnia. Therefore, we have designed a randomized controlled trial (RCT) involving a placebo control to ensure blinding of participants to investigate the effects of acupuncture on insomnia in improving sleep quality and psychosocial symptoms.

**Methods:**

We have designed a single-center, parallel-group, single-blinded RCT. A total of 252 participants who meet the eligibility criteria will be randomly allocated into a manual acupuncture group or sham acupuncture group in a 1:1 ratio. All participants will receive 24 sessions of acupuncture (30 min per session, three sessions per week for 8 weeks). Participants will be assessed using the Pittsburgh Sleep Quality Index score, self-assessment anxiety scale, self-assessment depression scale, and Medical Outcomes Study 36-Item Short-Form Health Survey at baseline and 8 weeks. All analyses will be based on an intention-to-treat principle. The results will be published in an international peer-reviewed journal.

**Discussion:**

The results of this study are expected to clarify the effects of acupuncture on sleep quality and psychosocial symptoms in patients with insomnia. This will contribute to the clinical practice of acupuncture in the management of insomnia.

**Trial registration:**

Chinese Clinical Trail Registry ChiCTR2100049172. Registered on 24 July 2021.

## Background

As a major public health problem, insomnia is defined as sleep difficulties including dissatisfaction with sleep quality, difficulty in initiating or maintaining sleep, and early morning waking, which can affect daytime functioning, and cause fatigue, depression, anxiety, and other symptoms [1, 2]. The prevalence of insomnia is 10–20%, and about 50% of cases involve chronic insomnia, which is defined as suffering from this condition for at least 3 nights each week for 1 month or more [3]. In China, the prevalence of insomnia is approximately 15% [4]. Sleep disturbance is thought to be closely associated with psychological disorders, including depression and anxiety [5]. A survey reported that nearly 30% of the population suffers from psychological disorders due to busy lives and high-pressure jobs, and most affected individuals experience different degrees of insomnia. However, insomnia has not yet been effectively resolved [6, 7].

Medications, such as antidepressants and melatonin, are the most commonly used treatments in the management of insomnia [8]. The use of sleeping pills is common among adults, and middle-aged women with higher education are more likely to have used anti-insomnia drugs [9]. However, adverse effects, including psychological and physical dependence, residual daytime sedation, and cognitive impairment, should not be ignored [10]. Therefore, the American Academy of Sleep Medicine and the American College of Physicians recommend non-pharmacological therapies, including acupuncture and relaxation, first-line treatment options for insomnia [11, 12].

Acupuncture is popular and safe traditional Chinese therapy. It has been used in the treatment of insomnia, depression, and anxiety in China for thousands of years. Acupuncture may restore physiological function through the insertion of needles to stimulate acupoints. Studies have indicated that acupuncture could be effective for sleep disorders by increasing the content of serotonin and aminobutyric acid [13], reducing glutamate levels [14], and improving the function of central inhibition [15]. With better sleep quality, depression and anxiety in patients with insomnia are improved. Previous reviews have shown that acupuncture affects insomnia, but there are no high-quality studies focusing on acupuncture for insomnia, especially for treating depression and anxiety due to insomnia [16, 17].

Therefore, it is important to conduct high-quality randomized controlled trial (RCT) to assess the effects of acupuncture on sleep quality, depression, and anxiety in patients with insomnia, with sham acupuncture as control. In this study, we designed a randomized, parallel-group, single-blinded trial to investigate the effects of acupuncture for insomnia in improving sleep quality and psychosocial disorders.

## Methods/design

### Study design

**Fig. 1:**
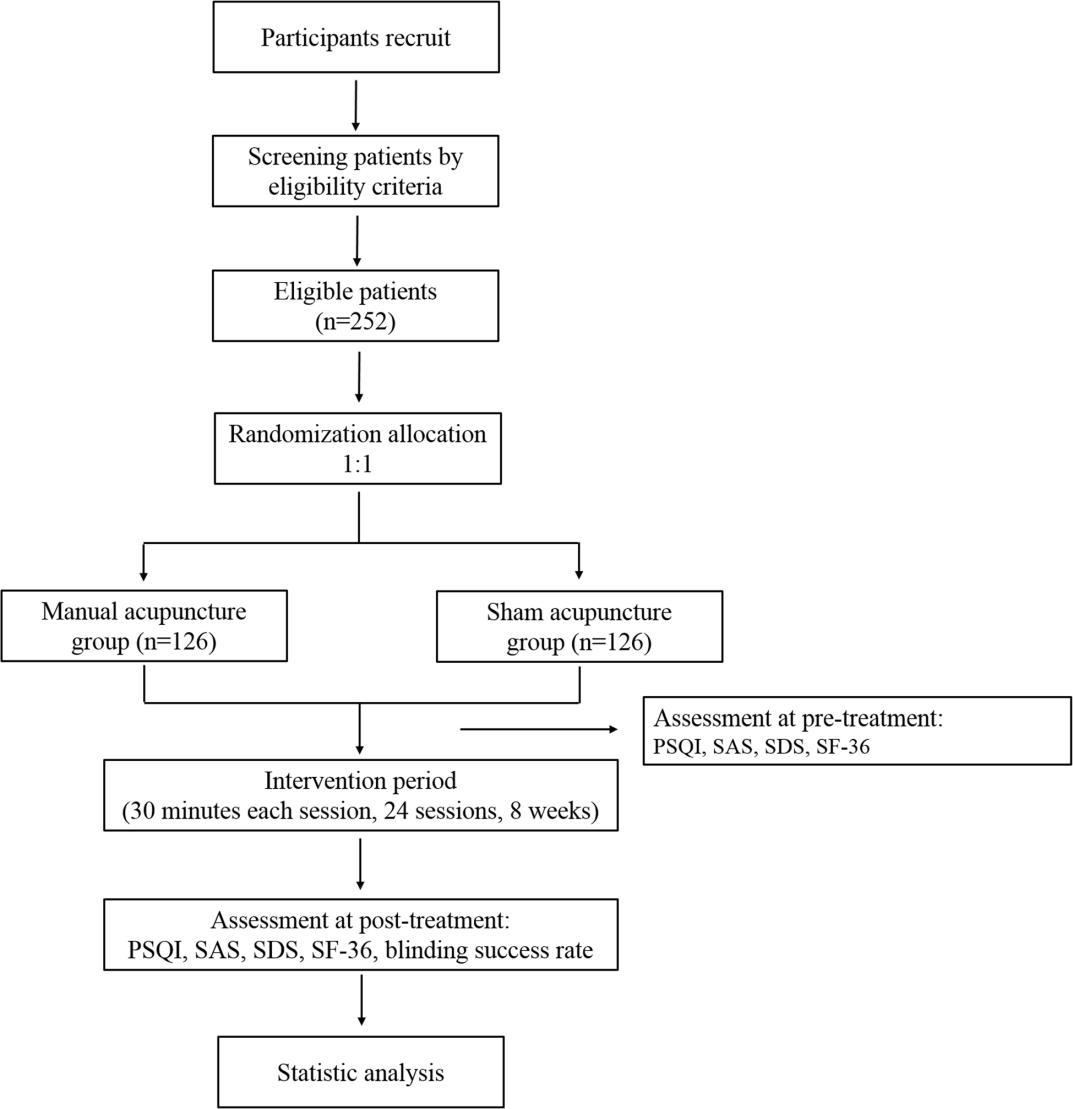
The study procedure

A single-center, parallel-group, single-blinded randomized controlled superiority trial has been designed to compare the effects of manual acupuncture and sham acupuncture in the treatment of insomnia. All participants will receive manual acupuncture or sham acupuncture during three sessions per week over 8 weeks. The study has been approved by the ethics committee of the Shaanxi Provincial Hospital of Chinese Medicine (2020-17). The study protocol follows the Standard Protocol Item Recommendations for Interventional Trials (SPIRIT) guidelines and Standards for Reporting Interventions in Controlled Trials of Acupuncture (STRICTA). This study protocol is funded by the Chinese Clinical Trials Registry. The registry number is ChiCTR2100049172, and the registration date is July 24, 2021. The study procedure is illustrated in Fig. 1. The trial schedule is listed in Table 1.

**Table 1:** The trial schedule

Items	Before enrollment (week − 1)	Intervention period (weeks 1–8)	End of intervention (week − 9)
Inclusion criteria	√		
Exclusion criteria	√		
Informed consent	√		
Baseline measurement	√		
Randomization and allocation	√		
PSQI	√		√
Quality of life	√		√
Depression	√		√
Anxiety	√		√
Blinding success rate			√
Adverse events			√

*PSQI* Pittsburgh Sleep Quality Index

### Participants

### Recruitment

Participants will be recruited from among outpatients of the Shaanxi Provincial Hospital of Chinese Medicine by advertisements on community boards and online social media (WeChat). Patients, who are interested in this study, will be asked to communicate with the researchers face-to-face for more detailed information including interventions, assessments, benefits gained from this trial, and potential adverse reactions. Before randomization, written informed consent will be obtained from all participants prior to their participation. Detailed attention, including trial procedure, intervention, benefits, and potential adverse events of participating in the trial, will be explained to potential participants before they provide informed consent. They will be instructed to provide informed consent after full consideration. The personal information of potential and enrolled participants will be collected and stored by the researchers, who will be required to sign a pledge to protect the confidentiality of the study participants.

### Inclusion criteria

The inclusion criteria are as follows:

1. Fulfillment of the diagnostic criteria for insomnia according to the Diagnostic and Statistical Manual of Mental Disorders [18];
2. Age 18–65 years (male or female);
3. History of insomnia of at least three times per week for more than 1 month;
4. No history of medication for improving insomnia or the medication must have been stopped for more than 3 weeks;
5. Psychological symptoms including anxiety (self-assessment anxiety scale [SAS] score ≥ 50) or depression (self-reported depression scale [SDS] score ≥ 50);
6. Agreement to participate in the trial and be randomly allocated into any of the study groups.

### Exclusion criteria

Participants who meet any of the following conditions will be excluded:

1. Presence of insomnia caused by nervous system diseases including stroke, Parkinson’s disease;
2. Presence of serious organic disease, or other chronic diseases, including cardiac disease, liver or kidney disease, or thrombocytopenia with a bleeding tendency;
3. Ongoing pregnancy or being a woman of childbearing age who is not on an appropriate method of birth control;
4. History of severe episodes of fainting during acupuncture or being afraid of blood;
5. Infection or inflammation at the acupoints;
6. Use of alcohol and/or other drugs or dependence on drugs.

### Sample size

Sample size estimation was based on changes in the Pittsburgh Sleep Quality Index (PSQI) score. According to the pre-trial, there was a mean difference in the PSQI of 2.0 with a standard deviation of 4.36 between the two groups. In the current study, we assume a significance level α of 0.05 and power (1-β) = 0.90, owing to which a sample size of 202 can provide effective power to reject the null hypothesis between the manual acupuncture group and sham acupuncture group using two-sided, two-sample *t*-tests assuming equal variance. Considering the expected dropout rate of 20%, this study will require at least 252 participants.

### Randomization and allocation concealment

A simple randomization method will be used in this trial. A total of 252 participants will be randomly assigned to either the manual acupuncture group (*n* = 126, expected) or sham acupuncture group (*n* = 126, expected) after the baseline assessment. The randomization sequence will be generated using Statistical Analysis Software 9.1 by a research assistant who will be blinded to the recruitment of participants. Randomization numbers will be sealed in a computer-generated randomization-based opaque envelope. Only the screening sequence number will be printed on the envelope. The researcher, who screens the eligible patients after baseline assessment, will separate the envelopes, and then assign the patient to the manual acupuncture group or sham acupuncture group.

### Blinding

The outcome assessors, data managers, and statistical analysts will be blinded to the treatment allocation. The manual acupuncture and sham acupuncture assignments will be blinded to the patients. The manipulation of needles and acupoints will be similar in both groups. To assess blinding, the participants will be asked whether they experienced manual acupuncture or sham acupuncture after the trial. It will be impossible to blind the acupuncturists because of the characteristics of acupuncture interventions. Unblinding will only be performed in cases of an emergency, such as any serious adverse event.

### Interventions

All participants will receive 24 sessions of acupuncture (30 min each session, three sessions per week for 8 weeks). Licensed acupuncturists, who have had more than 5 years of clinical experience in acupuncture therapy, will perform the acupuncture treatment. They are well-trained in identifying the locations of the acupoints and the manipulation of needles. The temperature will not be lower than 25 °C in the treatment room. Patients will be allowed to take 0.5–2 mg of Estazolam tablets (Approval number: H37023047, Shandong Xinyi Pharmaceutical Co., Ltd) orally before sleep as a rescue medication. The same acupoints will be used in both groups, including Shenting (GV24), Neiguan (PC6), Shenmen (HT7), Hegu (LI4), Anmian (EX-HN22), Zusanli (ST36), Sanyinjiao (SP6), Zhaohai (KI6), and Shenmai (BL62).

### Manual acupuncture treatment

The patients will lie on their backs in a relaxed position. After disinfection of the skin with 75% alcohol, sterile adhesive pads will be placed on the acupoints. Acupuncture needles 0.30 mm in diameter and 40 mm in length (Suzhou Hwato Medical Instruments Co. Ltd, China) will be used for manual acupuncture. The needles will be inserted through the pads for Deqi sensations, including pain, numbness, swelling, and heaviness sensation, indicating that the acupuncture is effective. The needles will then be maintained for 30 min. Manual stimulation will be conducted three times every 10 min. After 30 min, the needles will be removed, and pressure will be applied with clean cotton balls to avoid bleeding.

### Sham acupuncture treatment

The sham acupuncture group will receive non-insertive acupuncture using blunt-tipped placebo needles (0.30 mm in diameter and 40 mm in length, produced by Suzhou Hwato Medical Instruments Co. Ltd, China). The needles have a blunt tip that cannot penetrate the skin. The acupuncturist will choose acupoints, and the needle placement duration will be the same as for the manual acupuncture group. Sham manual stimulation will be conducted every 10 min. At the end of the treatment, the needles will be removed, and clean cotton balls will be pressed onto the acupoints, so that the patients feel a sensation similar to that of the withdrawal of real acupuncture needles.

### Discontinuation

The participants will be free to stop treatment and withdraw from this trial if they are unable or unwilling to complete the treatment, if the scientific research assistant suggests that it would be inappropriate for the patient to continue the study intervention due to a worsening state, or if adverse events, including subcutaneous hematoma, unfavorable or unintended, and fainting, occur during acupuncture treatment. In addition, patients will be advised to contact their doctors in the event of any undesirable effect after the application of acupuncture treatment. The doctor will diagnose and treat the adverse reactions and provide suggestions on whether to continue acupuncture treatment. The participants who are withdraw from the trial will still be followed up and complete the assessments of primary and secondary outcomes.

### Outcomes

#### Primary outcomes

The PSQI, a self-assessment questionnaire used to evaluate sleep quality, will be the primary outcome of this study. It will be administered at baseline and 8 weeks (at the end of the intervention). The PSQI assesses sleep based on seven domains: subjective sleep quality, sleep latency, sleep duration, sleep efficiency, sleep disturbance, hypnotic medication use, and daytime dysfunction. Each domain is rated from 0 to 3, so the total score of the PSQI ranges from 0 to 21. A cutoff score of 5 indicates insomnia [19].

#### Secondary outcomes

Secondary outcome measures refer to scores on the SAS, SDS, and Medical Outcomes Study 36-Item Short-Form Health Survey (SF-36), which will be assessed at baseline and 8 weeks (at the end of the intervention).

Psychological symptoms, including depression and anxiety, will be assessed using the SAS and SDS. The SAS is applicable to adults with anxiety symptoms and has a wide range of applications. The SDS can fairly directly reflect the subjective feelings of patients with depression and their changes in treatment. A higher total score indicates more severe anxiety and depression [20].

Quality of life will also be assessed as a secondary outcome, using the SF-36, which includes 36 items comprising eight categories: functional capacity (ten items), physical aspects (four items), pain (two items), general health status (five items), vitality (four items), social aspects (two items), mental health (five items), and a question comparing current health conditions with those from a year ago. The total scores range from 0 to 100. Higher scores indicate better health status [21].

Blinding success rate. All patients will undergo the blinding test twice, once each at 4 weeks and 8 weeks, to assess the success rate of participant blinding. The question “When you volunteered for the current trial, you were informed that you have an equal chance of receiving either manual acupuncture or sham acupuncture. Which one do you think you received?” will be asked to them. Patients will have the choice of answering with one of the following responses: manual acupuncture treatment, sham acupuncture treatment, or uncertain.

#### Data management

For the eligible participants, the researcher will collect baseline data at the appropriate time. The primary and secondary outcomes will be assessed by blinded assessors at baseline and 8 weeks (at the end of the intervention). Data will be collected in the case report form (CRF) by the assessors after obtaining signed consent from the patients. Two research assistants who will be blind to the allocation will double-enter and check the data from the CRF. To protect the privacy of the participants, codes and initials will be used instead of their personal information. All of the original data will be stored in ResMan Research Manager of the Clinical Trial Management Public Platform. The relevant research assistants will be trained to ensure that the system meets the trial requirements. In addition, the research manager will oversee clinical data management by monitoring it at least once a week.

#### Quality control

To insure high quality of the trial, all acupuncturists and assessors will undergo strict training before baseline data acquisition to guarantee consistent practices. The training will include diagnoses, inclusion and exclusion criteria, identification of the location of acupoints, acupuncture techniques, and completion of the CRF. The participants will be free to withdraw from the trial at any time for any reason to ensure data authenticity and credibility. Dropouts and withdrawals from the trial will be recorded to ensure an intention-to-treat (ITT) analysis.

In addition, an independent data and safety monitoring team will be established to identify problems, monitor and examine collected data, and control bias once a week. They have the right to suspend the trial or even terminate it at any point if they find any problems or adverse events during the trial. The monitoring team, who should declare no conflict of interest in this study, consists of three members with expertise in different fields: Professor Lili Wang in acupuncture from Shaanxi University of Traditional Chinese Medicine, Professor Liang Chen in statistics, and Professor Peng Jin in insomnia from Shaanxi Provincial Hospital of Chinese Medicine. If there are any problems with regard to the project, the monitoring team will make decisions to alter the details of this protocol and communicate them to the persons conducting the trial by written notice after approval by the application ethics committee.

#### Safety monitoring

Any adverse event, including subcutaneous hematoma, unfavorable or unintended, and fainting, related to acupuncture treatment will be reported by patients and researchers during each acupuncture treatment. In addition, all participants will undergo liver and kidney function examinations, routine blood tests, and stool tests to exclude severe heart/liver/kidney diseases and evaluate the possible side effects of the acupuncture.

#### Statistical analysis

Descriptive characteristics of baseline statistics will be reported as mean ± standard deviation or median. The primary and secondary outcomes will be analyzed based on the ITT. All qualified participants in both groups will receive the intervention and provide outcome assessment at least once. The missing data of participants who drop out will be replaced by the last observation carried forward’ method according to the ITT principle. A two-sample *t*-test or chi-square test will be used to test differences between the manual acupuncture group and sham acupuncture group using SPSS 19.0 by a blinded statistician. All planned tests are two-tailed. Statistical significance will be set at *P* ≤ 0.05.

### Discussion

The aim of the current trial is to investigate the effects of acupuncture on sleep quality and psychological disorders in patients with insomnia. Sleep quality will be assessed using PSQI scores. The focused psychological disorders in patients with insomnia will include depression (assessed by the SDS) and anxiety (assessed by the SAS). The overall quality of life of patients with insomnia will be assessed using SF-36. This study will attempt to associate the effects of acupuncture on sleep quality with the psychological effects of acupuncture. The study will provide evidence of the effects of acupuncture on patients with insomnia and depression or anxiety.

Depression and anxiety are the main consequences of chronic and long-term insomnia. These psychological symptoms ultimately degrade the quality of life of patients with insomnia. While the effects of acupuncture on insomnia have been investigated in previous studies [16, 17], this study will allow us to determine how to relieve insomnia symptoms by improving anxiety or depression, thus improving the quality of life of insomnia patients.

Although this trial was designed to address the limitations of previous studies, there are some limitations to the current study. First, it is difficult to design a double-blinded RCT because it is impossible to blind the acupuncturist. To minimize the risk of detection bias, the assessors and statistical experts will be blinded to the group assignment in this study. Second, the current study will use sham acupuncture as a manual acupuncture control based on STRICTA. However, sham acupuncture may lead to improvements in sleep quality and psychological symptoms in patients with insomnia. Third, patients inclined to use alternative therapies may be inclined to choose the manual acupuncture treatment, which may result in allocation bias during the recruitment of patients for sham acupuncture. In addition, there are no objective tools to assess sleep quality and psychological symptoms.

In summary, the current study will standardize acupoint selection, acupuncture manipulation, and outcome assessment by rigorously following the SPIRIT guidelines and STRICTA recommendations. We expect that this trial will confirm the effects of acupuncture on sleep quality and psychological symptoms in patients with insomnia. We also hope to provide a safe and effective treatment for insomnia.

## Trial status

The version number of this protocol is 2.0, dated on 18 June 2020. The study will be conducted in the Shaanxi Provincial Hospital of Chinese Medicine from January 2022 to February 2022. The clinical trial is currently recruiting participants.

## Data Availability

The results of this study will be published in peer-reviewed journals and posters or oral presentations at relevant conferences. All data will be available beginning 3 months and ending 3 years after publication of the results. The trial data will be available from the corresponding author upon reasonable request.

## Abbreviations

CRF: Case report form
ITT: Intention-to-treat
PSQI: Pittsburgh Sleep Quality Index
RCT: Randomized controlled trial
SAS: Self-assessment anxiety scale
SDS: Self-assessment depression scale
SF-36: Medical Outcomes Study 36-Item Short-Form Health Survey
SPIRIT: Standard Protocol Item Recommendations for Interventional Trails
STRICTA: Standards for Reporting Interventions in Controlled Trials of Acupuncture
